# Overcome the 60% passing score and improve the quality of assessment

**DOI:** 10.3205/zma000985

**Published:** 2015-10-15

**Authors:** Ara Tekian, John Norcini

**Affiliations:** 1 University of Illinois, College of Medicine at Chicago, Chicago, USA; 2 Foundation for Advancement of International Medical Education and Research , Philadelphia, USA

## Abstract

It is not unusual for institutions around the world to have fixed standards (e.g., 60%) for all of their examinations. This creates problems in the creation of examinations, since all of the content has to be chosen with an eye toward this fixed standard. As a result, the validity of the decisions based on these examinations can be adversely influenced, making them less useful for their intended purposes.

Over the past several decades, many institutions have addressed this problem by using standard setting methods which are defensible, acceptable, and credible [1], [2]. Many methods are available and the major reasons to use them is to ensure that test content is appropriately selected and to be as fair to the students and other test users as possible [2], [3].

One barrier to the wider use of these methods is that some institutions object to the fact that the fixed standard (e.g., 60%) has not been applied. However, it is possible to rescale the passing score so that it is equal to the fixed standard, and then apply that same rescaling calculation to all of the test scores. This ensures that the institutional guidelines are not violated and allows the application of accepted methods of standard-setting. In turn, the application of these methods allow the content of the test to be selected without regard to a fixed standard, increases the validity of the decisions being made, and ensures a fairer and more accurate test of students.

## Zusammenfassung

Es ist weltweit häufig üblich, formale Bestehensgrenzen (z. B. 60%) für alle Prüfungen festzulegen. Dies führt zu Problemen bei der Entwicklung von Prüfungen, da der gesamte Inhalt der Prüfung im Hinblick auf diesen festgelegten Standard abzustimmen ist. Infolgedessen kann die Validität der Entscheidungen, die auf diesen Prüfungen fußen, nachteilig beeinflusst werden und den Nutzen für ihren beabsichtigten Verwendungszweck einschränken.

Im Laufe der letzten Jahrzehnte begegneten viele Institute diesem Problem durch die Verwendung von Verfahren des „Standard-Settings“, also Festlegungen der Bestehensgrenzen, die vertretbar, akzeptabel und zuverlässig sind [1], [2]. Hierzu steht eine Vielzahl an Methoden zur Verfügung und die Hauptgründe, diese zu nutzen, bestehen darin, sicherzustellen, dass der Testinhalt adäquat ausgewählt und so fair wie möglich gegenüber den Studierenden und anderen Testanwendern ist [2], [3].

Ein Hindernis für die breitere Anwendung dieser Verfahren ist, dass vielerorts die Tatsache beanstandet wird, dass dabei die formal festgesetzte Bestehensgrenze (z. B. 60%) nicht eingehalten wird. Es ist jedoch möglich, durch Umskalierungen die Bestehensgrenze neu zu definieren, so dass diese dem festgelegten Standard entspricht und dieselbe Skalierung auf alle Testergebnisse anwendet. Dies stellt sicher, dass die institutionellen Richtlinien nicht verletzt werden und ermöglicht die Anwendung der anerkannten Verfahren des Standard-Settings. Im Gegenzug gestattet die Anwendung dieser Verfahren die Auswahl der Prüfungsinhalte ohne Berücksichtigung des festgelegten Standards, was die Validität der getroffenen Entscheidungen erhöht und eine gerechtere und genauere Prüfung der Studierenden ermöglich.

## Commentary

Over the past several decades, many authors have advocated for setting standards for passing a test which are defensible, acceptable, and credible [1], [2]. Many methods are available and the major reasons to use them is to ensure that test content is appropriately selected and to be as fair to the students as possible [2], [3]. 

The implementation of a standard setting method moves the passing standard away from a fixed number (i.e., 60%) to a cut score which can vary depending on the difficulty of the test. For example, when two forms of an exam are administered, where Form A is slightly more difficult than Form B, setting the same passing standard for both will give an unfair advantage to students taking Form B – this threatens the validity of the test, by passing candidates who may not be qualified simply because of the test characteristics (difficulty) of the exam. Setting a relative passing standard (e.g., mean – 2 x standard deviation) does not overcome this problem, because the ability of students can change from year to year. Application of an absolute standard-setting method that relies on the judgments of a panel of expert is preferred.

Examinations may be administered for formative and summative purposes. Formative assessment is focused on providing feedback to the students. However, summative examinations which are focused on making decisions about students’ competence might have a significant impact on a student’s career pathway. 

In all of the health professions, including medicine, the undergraduate students need to be judged on their mastery of their professional content. They need to be competent in terms of knowledge and performance, and therefore, need to be assessed against a set of criteria or standards. The standard setting processes utilized for such purposes generate absolute standards, in contrast to relative standards where students are judged against each other. These standards are considered absolute, because they are expressed in terms of how much content the students need to know and thus theoretically all could pass or fail. Therefore, the success rate for any examination might vary depending on the passing score established by content experts. 

There are various methods of setting absolute standards, and the judgments might be focused on either the items, know as item-based (Angoff, Ebel [4], [5]), or on the examinee, that is examinee-based (Borderline or Contrasting group methods [6]). There are also compromise methods of setting standards, for example the Hofstee method, where judgments about how much needs to be known are combined with the relative performance of the students, also popularly known as the relative-absolute compromise method [7]. The passing scores might vary depending on the choice of method [2], [8]. 

In some methods, the judgments underlying standard setting are based on the definition of a hypothetical borderline student who would have a 50%-50% probability of passing the exam. The description of the borderline student is based on a consensus definition in the content areas represented by the blueprint and generated by the expert panel. This definition is based on a hypothetical candidate who on a given day would pass the exam and on a different day, fail the exam; the competency demonstrated by this candidate should represent uncertainty about the qualifications and attributes required for a passing candidate; it could also include descriptions of “forgivable” qualities that the candidate may not yet have, but over the course of his or her training, continue to master. 

Although the Angoff method for standard setting is a very popular method used in many medical schools, attention must be paid to the selection of judges, since their level of expertise, and the ability to answer the exam items correctly may affect the passing score [9]. Choice of credible judges and their calibration are equally important in standard setting, particularly during discussions of the borderline-examinee, and during training sessions/ exercises where the probability of a borderline-examinee answering or performing a checklist item correctly is being estimated [2].

Some institutions have started using the Angoff or other methods to set the passing score for OSCE examinations. They have adopted this strategy for OSCE examinations because the institution will not notice or object that the 60% passing score is not applied, or they have manipulated the difficulty of the OSCE examination in such a way as to have a passing score of 60% without any adjustments. However, the results of a standard setting exercise can also be rescaled to the passing score determined by the university, such as 60%. This can be achieved, without violating the institutional guidelines, by converting the raw passing score of an exam, determined through standard setting into a rescaled passing standard of 60% [10]. The scores of students are then rescaled as well. For example, if the passing score using the Angoff method for an MCQ examination was calculated to be 54%, that score is rescaled to 60% and then the examination is rescaled as well, so that the students and passing score are consistent with the ‘60%’ policy. Therefore, following a standard setting method will overcome the capricious nature of assigning a passing score of 60%, by simply transposing the true passing score to the institutionally required cut-off score. 

There is no “gold standard” for setting passing scores [11], [2]. Usually the choice of the standard setting method is based on the available resources and the practical realities of the educational environment. As such, it is critical to document all procedures used in establishing the passing standard, especially in a language the school is willing to make public. Every effort should be exerted to use one of the standard setting approaches described in the literature so as to make the passing scores defensible and meaningful. To accomplish this, there needs to be intensive faculty development, so that everyone understands the importance and consequences of setting standards, and are trained and calibrated to set passing scores. Moreover, faculty selected to participate in the standard setting process need to be representative and acceptable to the stakeholders. This may also require institutional change and one way to accomplish this is to hold meaningful discussions with institutional leaders about the rationale for standard-setting and evidence behind it. Using standard setting procedures necessitates changing the institutional assessment culture and promoting fairness and justice in measuring the competence of the students.

## Competing interests

The authors declare that they have no competing interests.
